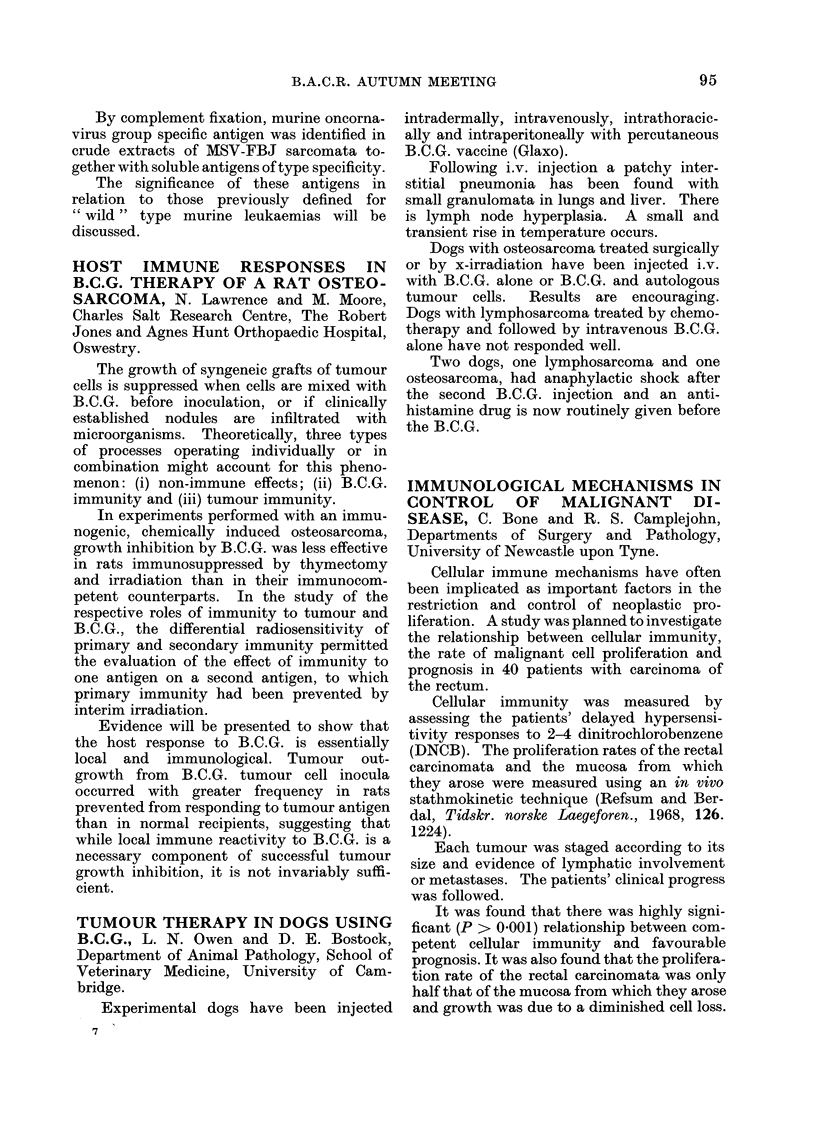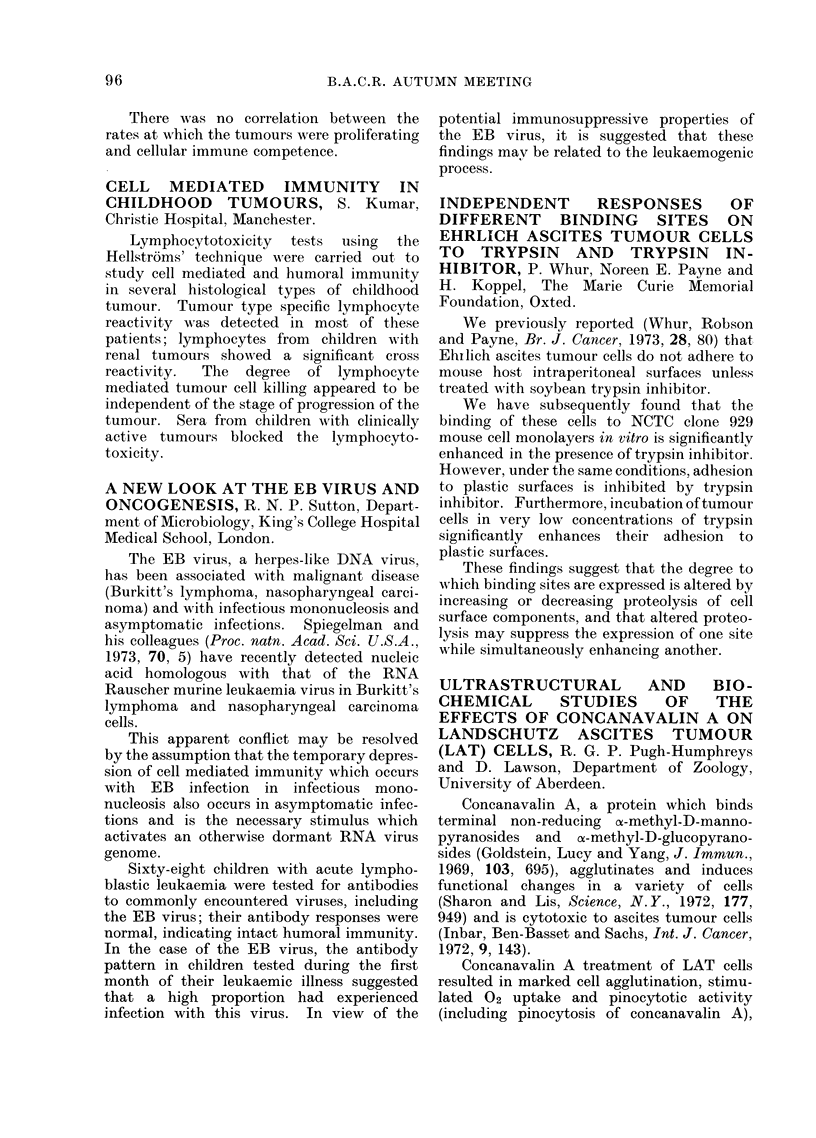# Proceedings: Immunological mechanisms in control of malignant disease.

**DOI:** 10.1038/bjc.1974.25

**Published:** 1974-01

**Authors:** C. Bone, R. S. Camplejohn


**IMMUNOLOGICAL MECHANISMS IN CONTROL OF MALIGNANT DISEASE,** C. Bone and R. S. Camplejohn, Departments of Surgery and Pathology, University of Newcastle upon Tyne.

Cellular immune mechanisms have often been implicated as important factors in the restriction and control of neoplastic proliferation. A study was planned to investigate the relationship between cellular immunity, the rate of malignant cell proliferation and prognosis in 40 patients with carcinoma of the rectum.

Cellular immunity was measured by assessing the patients' delayed hypersensitivity responses to 2–4 dinitrochlorobenzene (DNCB). The proliferation rates of the rectal carcinomata and the mucosa from which they arose were measured using an *in vivo* stathmokinetic technique (Refsum and Berdal, *Tidskr. norske Laegeforen.*, 1968, **126**. 1224).

Each tumour was staged according to its size and evidence of lymphatic involvement or metastases. The patients' clinical progress was followed.

It was found that there was highly significant ($P > 0{\cdot}001$) relationship between competent cellular immunity and favourable prognosis. It was also found that the proliferation rate of the rectal carcinomata was only half that of the mucosa from which they arose and growth was due to a diminished cell loss.

There was no correlation between the rates at which the tumours were proliferating and cellular immune competence.